# Takayasu's Arteritis: A Case Report

**DOI:** 10.31729/jnma.7685

**Published:** 2022-12-31

**Authors:** Arzoo Khadka, Sumi Singh, Sarika Timilsina

**Affiliations:** 1 Department of Internal Medicine, Nepal Medical College and Teaching Hospital, Jorpati, Kathmandu, Nepal; 2 Nepal Police Hospital, Maharajgunj, Kathmandu, Nepal

**Keywords:** *aortitis syndrome*, *arteritis*, *case reports*, *pulseless disease*, *young female arteritis*

## Abstract

Takayasu's arteritis is a chronic vasculitis of medium and large vessels. The most involved vessel is the aorta and its major branches. The disease is primarily seen in young women. The described incidence of the disease ranges from 0.3 to 3.3 million per year. The vessels are characterized by mononuclear infiltration and granulomatous inflammation of vascular media, which leads to arterial wall thickening with stenosis, occlusion, and aneurysmal dilation. Here we present a case of Takayasu's arteritis in a 26-year-old woman who presented with syncope and dizziness with thickened walls of the arch of the aorta and its branches in Magnetic Resonance Imaging angiogram finding. Women of Japanese descent are not the only ones who can develop Takayasu's arteritis; it can affect anyone. Therefore, early diagnosis and treatment are warranted. When the disease is dormant, the outcome seems favourable.

## INTRODUCTION

Takayasu is a rare kind of chronic inflammatory vasculitis that mainly affects Asian women under the age of 40. The prevalence mentioned varies between 4.7 and 360 instances per million.^1^ The exact aetiology is assumed to be a cell-mediated inflammatory process within the vasculature, which can result in occlusion, aneurysmal dilatation, and constriction in afflicted segments because of mononuclear and granulomatous infiltrates, which causes a variety of symptoms.^2^ During the disease's acute phase, patients may also have symptoms such as limb weakness or pain, headaches, syncopal attacks, and uneven blood pressure. Fever, weight loss, and exhaustion are examples of constitutional symptoms that may occur first.

## CASE REPORT

A 26-year-old woman who experienced frequent syncopal attacks and lightheadedness was hospitalized at our hospital. Initially, just dizziness preceded the syncope; but, as time went on, there were also brief instances of loss of consciousness after the syncope. She also expressed concerns about myalgia, decreased appetite, and weariness. Over a year of symptoms were present, and 2 months before admission, they relapsed. Physical examination revealed a striking difference in blood pressure between the right and left arms. Her systolic blood pressure in the right arm was between 80-90 mm of Hg, but her diastolic blood pressure was undetectable. She had impalpable brachial and radial pulses on her right. Her body temperature was 38.3°C, and her heart rate was 110 beats per minute. Exams of the chest and precordial areas were clear. Trans-thoracic echocardiography, electrocardiogram (ECG), and chest radiography performed afterwards showed no abnormalities. Low haemoglobin levels (11.9 g/dl), an increased erythrocyte sedimentation rate (51 mm/ hr), and low levels of C-reactive protein (28.1 g/dl) are all positive laboratory findings. Blood cultures, TB skin and sputum tests, laboratory tests for sexual diseases, and autoimmune serological results all came back negative.

**Figure 1:**
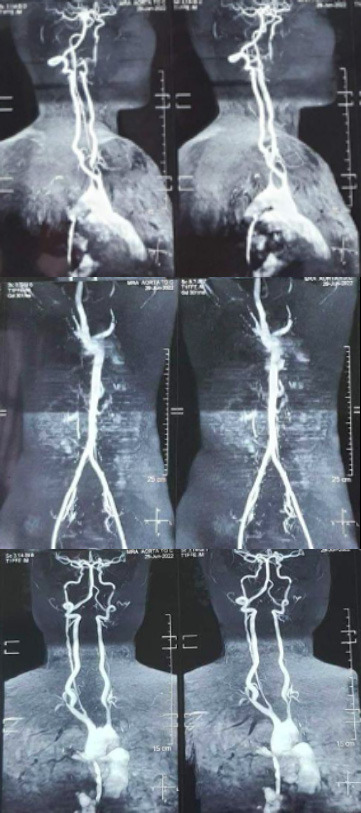
MRI scan showing thickening of the arch of the aorta and narrowing of the lumen of bilateral subclavian arteries.

Magnetic resonance imaging (MRI) angiography of the aorta and its branches was carried out, and the results showed aberrant thickening of the aortic arch's walls, which had a diameter of roughly 4.4 mm. The brachiocephalic, right subclavian, right common carotid, left common carotid and left subclavian branches of the aorta all exhibited thickened walls. With no flow signal within the lumen of the mid and distal parts, there was significant bilateral constriction in the subclavian artery lumen. The dorsal aorta, abdominal aorta, and other branches from the aortic arch were all clear and of typical size. Consequently, it was determined that the patient had takayasu arteritis in an active phase (Figure 1).

Prednisolone 40 mg/day orally, methotrexate 10 mg/week, aspirin 75 mg/day, and folic acid 5 mg/week were used to treat her. Within two weeks, inflammatory markers returned to normal, and she was allowed to leave the hospital. On her follow-up visit, the patient reported clinical improvement and continued to be symptom-free. A maintenance dose of 12 mg/day of methylprednisolone was gradually decreased. The patient is still asymptomatic 6 weeks after the follow-up visit.

## DISCUSSION

After the Japanese ophthalmologist Mikito Takayasu, who first documented a case in 1905, Takayasu arteritis is so named.^3^ The aorta, its major branches, the coronary and pulmonary arteries, as well as other big and medium-sized vessels, are all affected by the chronic inflammatory vasculitis known as Takayasu. Because of the frequent obstruction of big arteries coming from the aorta, it is also known as a pulseless condition or occlusive thromboaortopathy. The exact pathophysiology is unknown.^4^ However, pan arteritis, which has substantial intimal hyperplasia, medial and adventitial thickening, infiltration of mononuclear cells, and occasionally giant cells, is thought to be the cause.^5^ It is mostly observed in Asian-descent women, peaking in the 30s. Takayasu illness affects 2 in 10,000 individuals annually, with a male to female ratio of 8:1.6 Takayasu's national data are still not available, though.

In the case study done by Manfrini O and Bugiardini R, a 54-year-old Caucasian female was admitted for pulmonary edema and she was a known case of hypertension.^7^ In our case, the patient presented with frequent syncopal attacks and lightheadedness. All her medications were stopped as she had an episode of syncope and low blood pressure. On further examination, there was blood pressure discrepancy on her bilateral hands and there was an increase in the acute phase reactant level.^7^ On the angiographic findings, there was focal narrowing of of the abdominal and thoracic aorta as well as severe calcification in the whole aorta. Two stents were placed, and she was treated with oral methylprednisone, cloptidogrel, and diuretics.^7^ On the 6 months follow-up, the patient was asymptomatic. In our study, prednisolone 40 mg/day orally, methotrexate 10 mg/week, aspirin 75 mg/day, and folic acid 5 mg/week were used to treat her.

Depending on the vessels involved, the takayasu disease can manifest clinically in a variety of ways. It typically begins with constitutional symptoms like fever, weight loss, claudication, and fatigue, and then progresses to other symptoms like weakness, lightheadedness, dizziness, high blood pressure, retinopathy, aortic regurgitation, vascular bruits, neurological symptoms like seizures and syncope, etc., depending on the occlusion caused by the inflammatory infiltrates. In almost 20% of instances, neurological symptoms are present.^8^ vessels were involved in the distribution of involvement.

Three out of the six criteria listed above have a sensitivity of 90.5% and a specificity of 97.8% for diagnosis.^9^

The conditions atherosclerotic, inflammatory, infectious, and genetic that affect the major arteries are included in the differential diagnosis of Takayasu disease. Examples include atherosclerosis, fibromuscular dysplasia, TB, and giant cell arteritis. The gold standard for diagnosing Takayasu's Arteritis is angiography. Doppler and non-invasive MRA, however, can also produce just as good results.^10^

Acute phase reactants like ESR and CRP also provide additional evidence in favor of the diagnosis. The mainstay of treatment is expected to be systemic glucocorticoids and immunosuppressants, which are believed to reduce inflammation and limit the development of the illness.^11^ If irreversible arterial stenosis develops due to conditions such cerebral ischemia, hypertension with critical renal artery stenosis, extremity claudication, or both, surgical intervention (endovascular) may be required.^12^ To avoid problems, surgical intervention is typically discouraged during active disease and advocated during quiescent disease.

Our patient is under 40 years old, has bilateral brachial pulse impalpability, significant lumen narrowing of the bilateral subclavian arteries, abnormal thickening of the wall of the arch of the aorta, brachiocephalic artery, right subclavian artery, and right as well as left common carotid artery, all of which meet the diagnostic criteria. Collateral vessels sustain the brain perfusion. Syncope is a brief loss of consciousness brought on by cerebral hypoxia. Our patient experienced dizziness before sporadic syncope.

This case study is one of the few studies performed on the subject of takayasu's arteritis in Nepal as the disease is rare and mostly affects East Asian women under the age of 40. However, the patient in this case was only present in the follow-up for 6 weeks and her long-term conditions are not present on the study. It is recommended that the patient of takayasu's arteritis must be observed for long-term, even after being discharged from the hospital.

Takayasu's arteritis mainly affects large vessels and its branches. However, all the large vessels are not affected at once and the symptoms are in correspondence with the vessels affected. As seen in the case, there was a significant narrowing of right as well as left subclavian artery, and the patient was present with headache, dizziness and syncopal attacks. The patient experienced dizziness before sporadic syncope, which is because there was significant narrowing of aorta and its branches causing blood pressure discrepancy.
